# The Physical Side of the Probationer's Training

**Published:** 1919-08-09

**Authors:** 


### THE MATRONS' AND SISTERS' DEPARTMENT.

### THE PHYSICAL SIDE OF THE PROBATIONER'S TRAINING.

It is always an interesting thing to compare a group of candidates before their preliminary training with the same young women after six weeks or so of intensive preparation for their entrance into the wards. The nervous, bumptious, or self-satisfied individual has begun to lose her uneasy consciousness of self. She has set about acquiring the right manner, and her attitude has changed towards herself as well as towards those about her. Silent, composed, outwardly self-reliant, the little company stands awaiting the final ordeal of approval by the matron. The seal of their chosen profession has already marked them for its own.

The system which prepares both mind and body before admitting the probationer to the hospital is likely to spread and become universal. And the fact brought most conspicuously to light in preliminary training-schools is the extent to which harmony of mind depends on the harmonious movements of the body. When that truth is fully recognised in all hospitals a wonderful change will come over the nursing profession. No longer will nurses be suffered to incur, through neglect of their own bodies, diseases which the hospital exists to cure. No longer shall we behold in every gathering of nurses startling examples of bad carriage and the defects proceeding therefrom. Who has not noted among nurses examples of stooping shoulders, of shambling gait, of head poked forward, of stiff and angular action of the arms, which training has neglected to correct? Truth to tell, nurses often lack that freedom of gait and graceful action of the limbs which spring from perfectly co-ordinated movements of the body. Perfect physical well-being is so essential to healers that it is worth enquiring whether the poise which speaks a balanced mind could not be imparted to the women who pass three or four years in institutions possessing every facility for giving this kind of training.

Very few probationers have had any satisfactory physical instruction before commencing their hospital life. A few perfunctory calisthenics at school sum up their advantages in this respect. Yet probationers enter upon hard tasks. They are called upon to meet demands upon their endurance, which probably exceed anything in their previous experience. They need to be very fit to encounter their tasks with ease. In short, they need physical preparation for their new life.

At St. Thomas's Hospital the experiment was tried of affording the nurses daily a few minutes' physical exercises under a competent instructress, and we understand that the result was most excellent. While never compulsory, the exercises were popular and were found to have a good effect in reducing fatigue. Considering the short space of time involved, the effect on the energies of those who systematically use Swedish exercises is remarkable. Regular instruction for no more than a quarter of an hour suffices to impart the secret of self-domination. Few women without such help know how to breathe, to stand, to walk, to run, to leap, to stretch their limbs in such a way as to stimulate vitality and make the best of their bodies. It would not be amiss if they learned to dance, to curtsey, to come into a room and leave it with dignity, to march in procession, in short, to hold up their heads wherever placed. Nurses are always the most conspicuous persons on the scene of action. Not a few are terribly worried by self-consciousness all their lives, and actually shrink from promotion on account of unconquerable nervousness. All this is swept automatically away by a few months of regular physical exercises. Even examination fever is almost purely physical. Half the trials from which nurses suffer in strange surroundings, working with unknown doctors, called out continually to startling patients and their even more startling friends, could be eased if not altogether abolished by that serene poise which gives the mastery over every bodily impulse.

There is a spiritual beauty in harmonious movements of the body. Assuredly we love many whose angularities distress us in spite of ourselves. Yet in healing others, no precaution is too minute to take which may remove impediments from the patient's path. It is exceedingly difficult to teach some probationers to be gentle, to move silently, to avoid brusque actions. Some even fail altogether, and are dismissed during their trial month because they are "rough." Would it not be easier for the ward sister and far, far, easier for them if they were shown the way to make their wrists supple, and their hands sure, their feet invisible and their backs straight, side by side with the formal training in matters which may be more important, but are certainly not thrust more continuously under notice?

